# Supplementary material for: Third Generation Buchwald Precatalysts with XPhos and RuPhos: Multigram Scale Synthesis, Solvent-Dependent Isomerization of XPhos Pd G3 and Quality Control by 1H- and 31P-NMR Spectroscopy
Source: Molecules. 2021 Jun 9;26(12):3507. doi: 10.3390/molecules26123507 (PMC8228727; doi:10.3390/molecules26123507)
Supplement: Supplementary file 1 [file molecules-26-03507-s001.zip › molecules-1224276-supplementary.pdf]

Supplementary Materials

The third generation Buchwald precatalysts with XPhos and RuPhos: multi-gram scale synthesis, solvent-dependent isomerization of XPhos Pd G3 and quality control by ^{1}H and ^{31}P NMR spectroscopy

Svitlana O. Sotnik [1, 2, 3], Artem M. Mishchenko [1, 4], Eduard B. Rusanov [5], Andriy V. Kozytskiy [1,2], Konstantin S. Gavrilenko [1,3], Sergey V. Ryabukhin [1,3], Dmitriy M. Volochnyuk [1, 3, 5], Sergey V. Kolotilov [2,3*]

[1] *Enamine Ltd, 78 Chervonotkatska str., Kyiv 02660, Ukraine*; sotniksvitlana@ukr.net (S.S.A.); kgavrio@gmail.com (K.S.G.)
[2] *L.V. Pisarzhevskii Institute of Physical Chemistry, National Academy of Sciences of Ukraine, Nauky Ave. 31, Kiev 03028, Ukraine*; s.v.kolotilov@gmail.com (S.V.K.); kozytskiy@gmail.com (A.V.K.)
[3] *National Taras Shevchenko University of Kyiv, 60 Volodymyrska str., Kyiv, 01033, Ukraine*; s.v.ryabukhin@gmail.com (S.V.R.)
[4] *V.I. Vernadsky Institute of General and Inorganic Chemistry, National Academy of Sciences of Ukraine, Palladina Ave. 32/34, Kiev 03142, Ukraine*; a.m.mishchenko@ukr.net (A.M.M.)
[5] *Institute of Organic Chemistry, National Academy of Sciences of Ukraine, Murmanska Str. 5, Kiev 03028, Ukraine*; XRAY@ioch.kiev.ua (E.B.R.); d.volochnyuk@gmail.com (D.M.V.)
* Correspondence: s.v.kolotilov@gmail.com (S.V.K.)

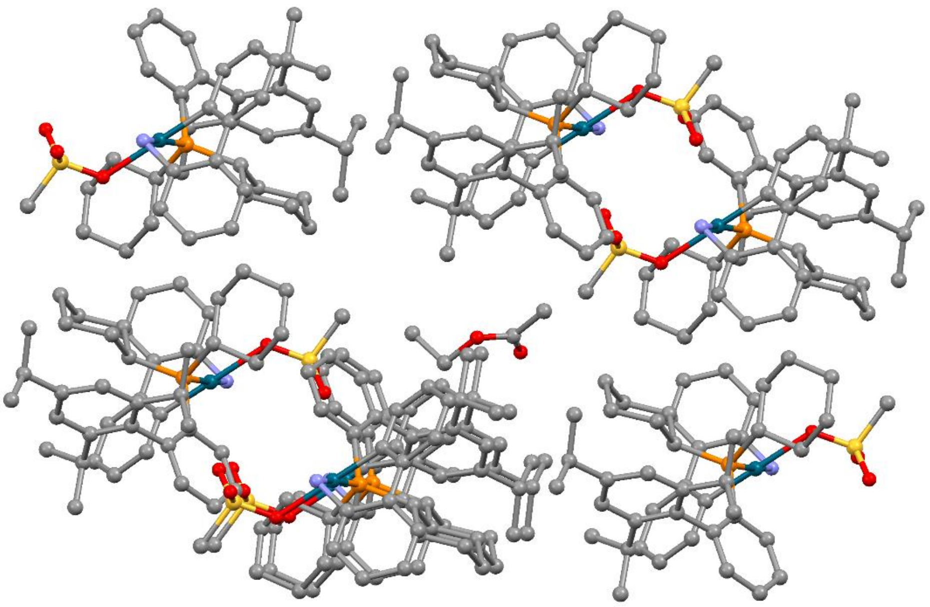

Figure S1. Fragment of crystal packing of **3a** showing the environment of captured ethyl acetate

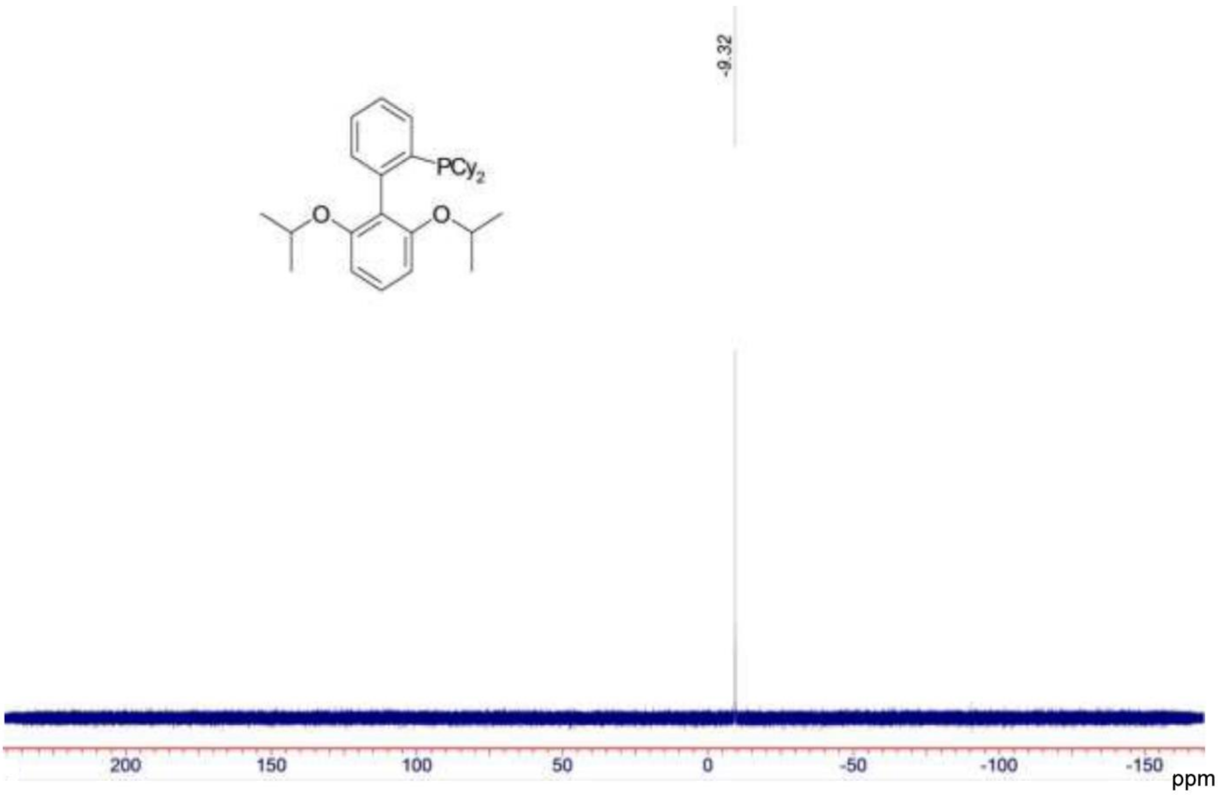

Figure S2. ^{31}P NMR spectra of RuPhos in THF.

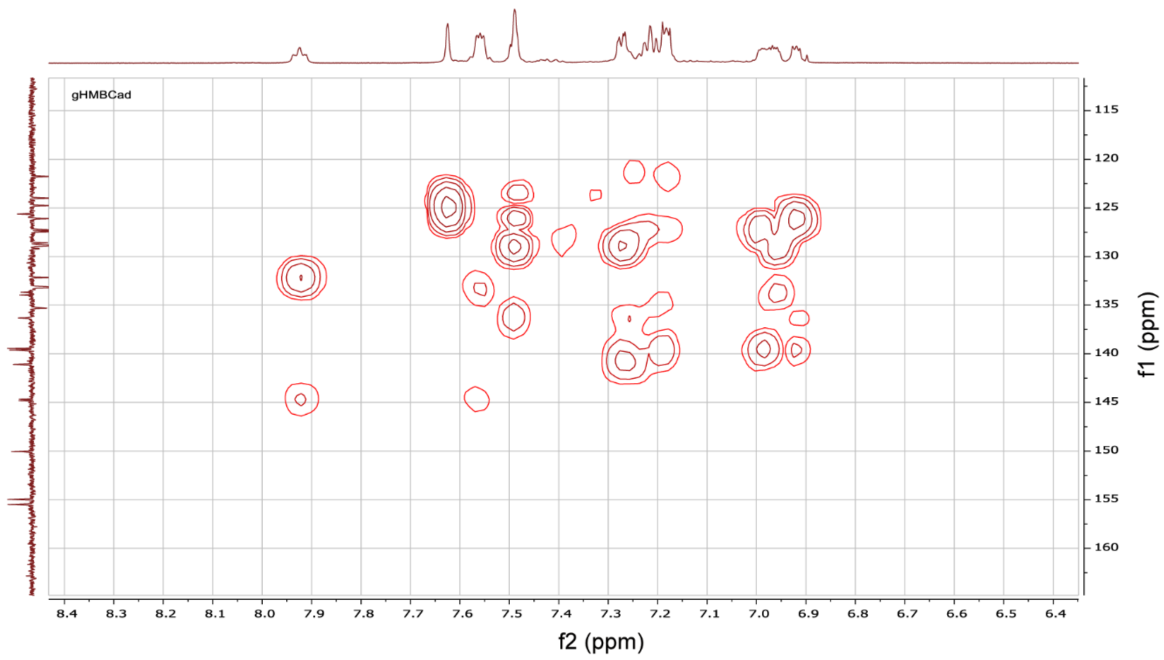

Figure S3. ^{1}H-^{13}C HMBC of XPhos Pd G3 in DMSO-d_6 solution.

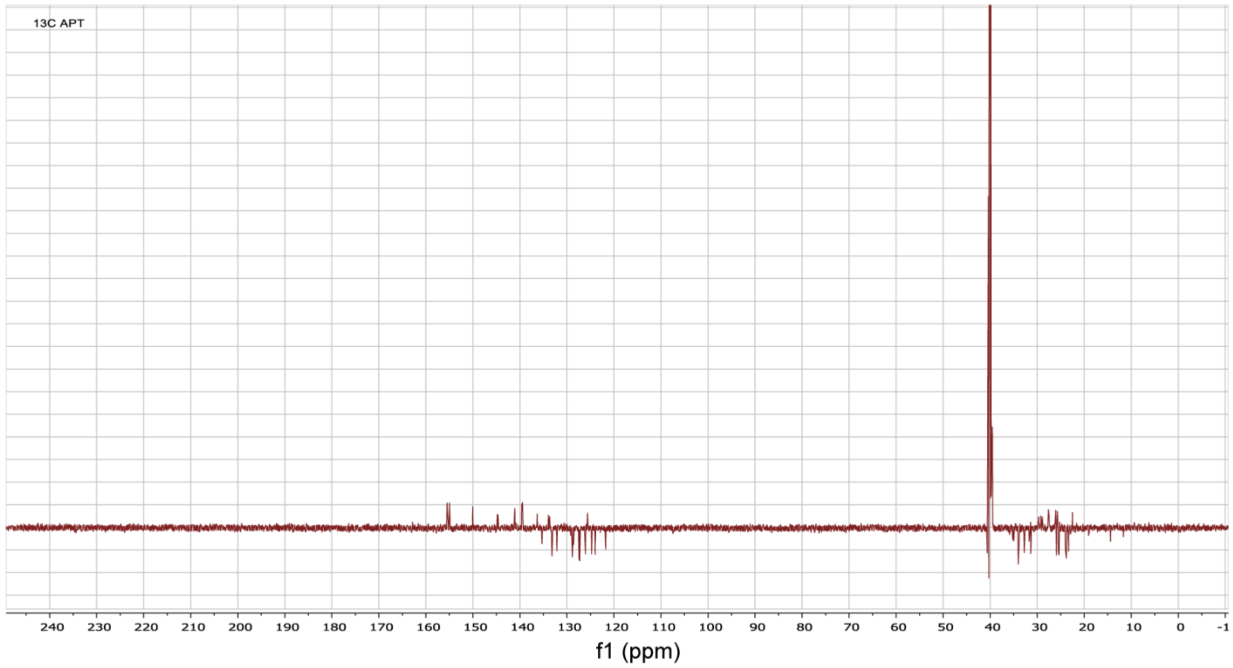

Figure S4. ^{13}C APT of XPhos Pd G3 in DMSO-d_6 solution.

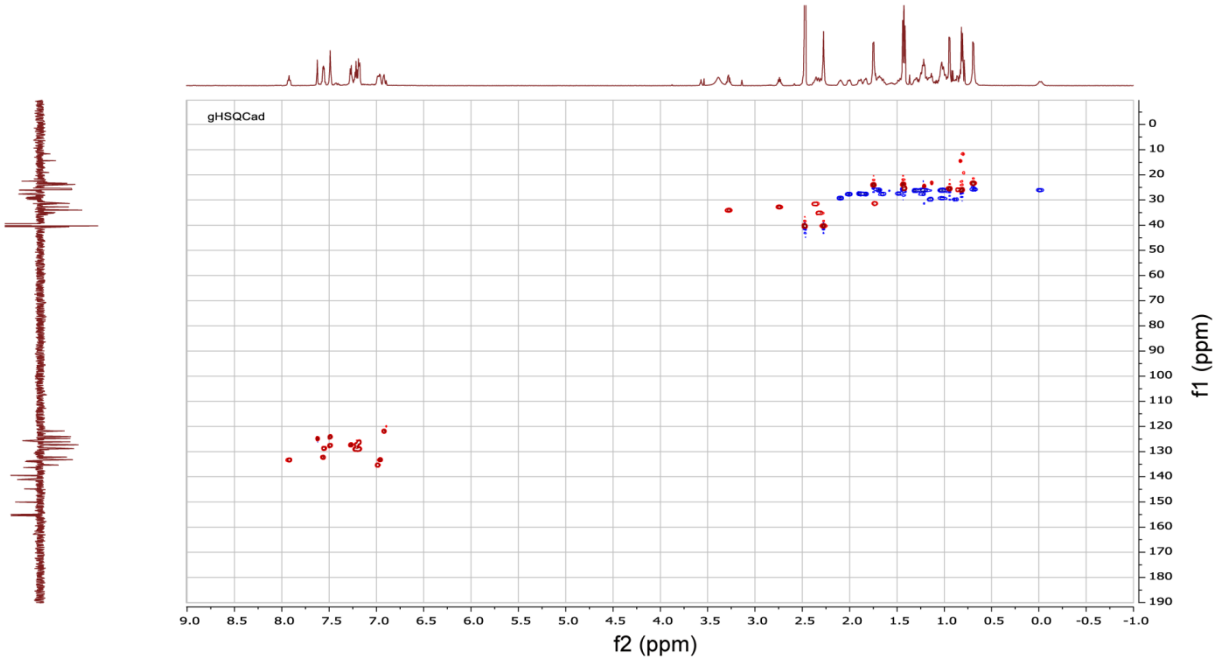

Figure S5. HSQC of XPhos Pd G3 in DMSO-d_6 solution.

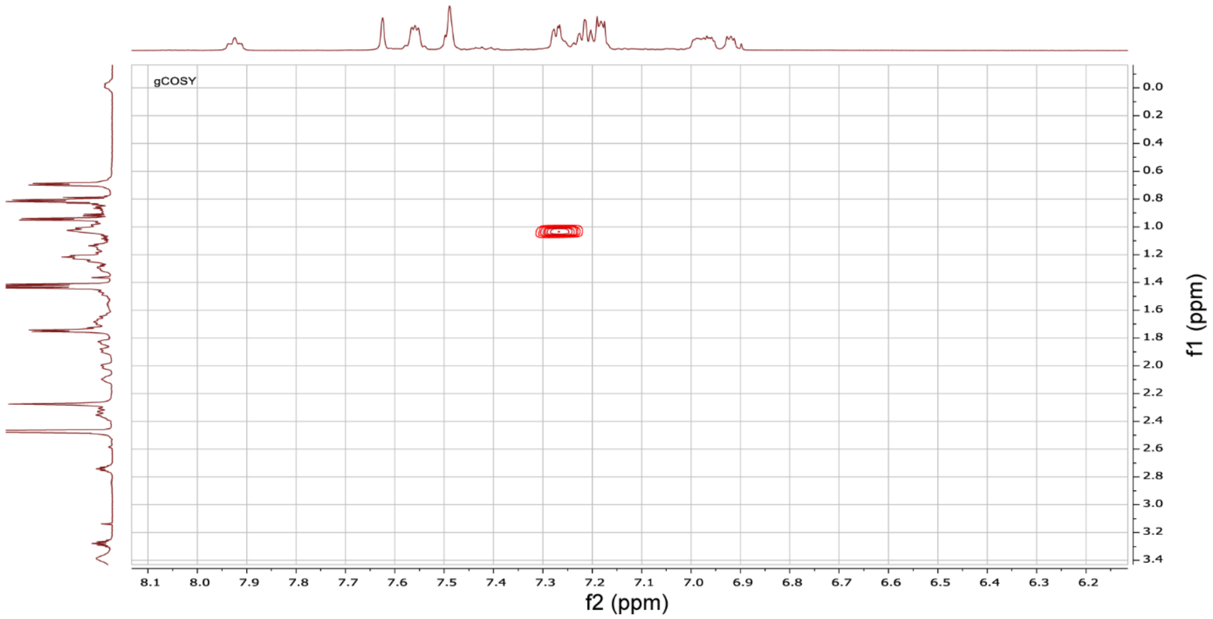

Figure S6. COSY of XPhos Pd G3 in DMSO-d_6 solution.

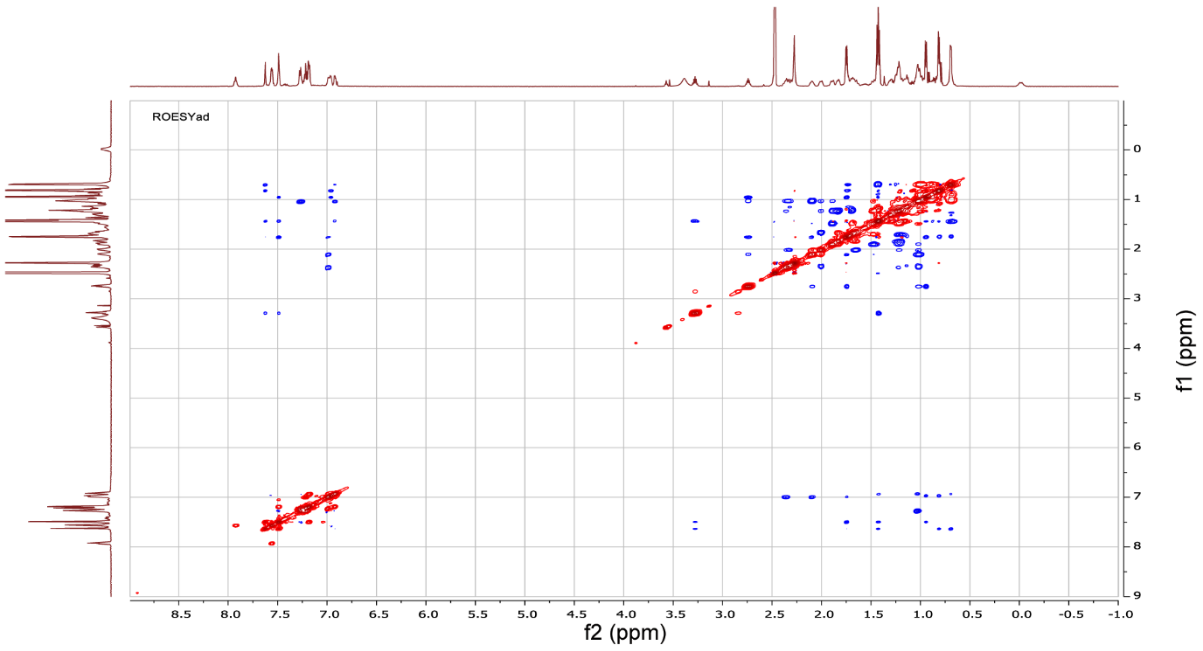

Figure S7. ROESY of XPhos Pd G3 in DMSO-d_6 solution.

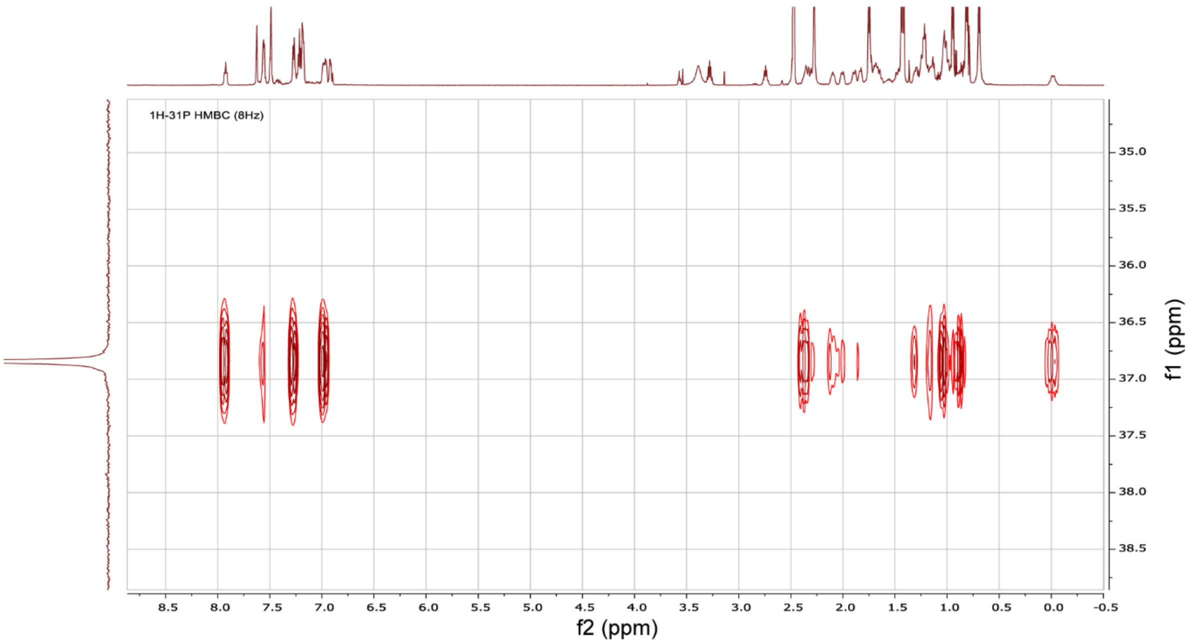

Figure S8. ^{1}H-^{31}P HMBC of XPhos Pd G3 in DMSO-d_6 solution.

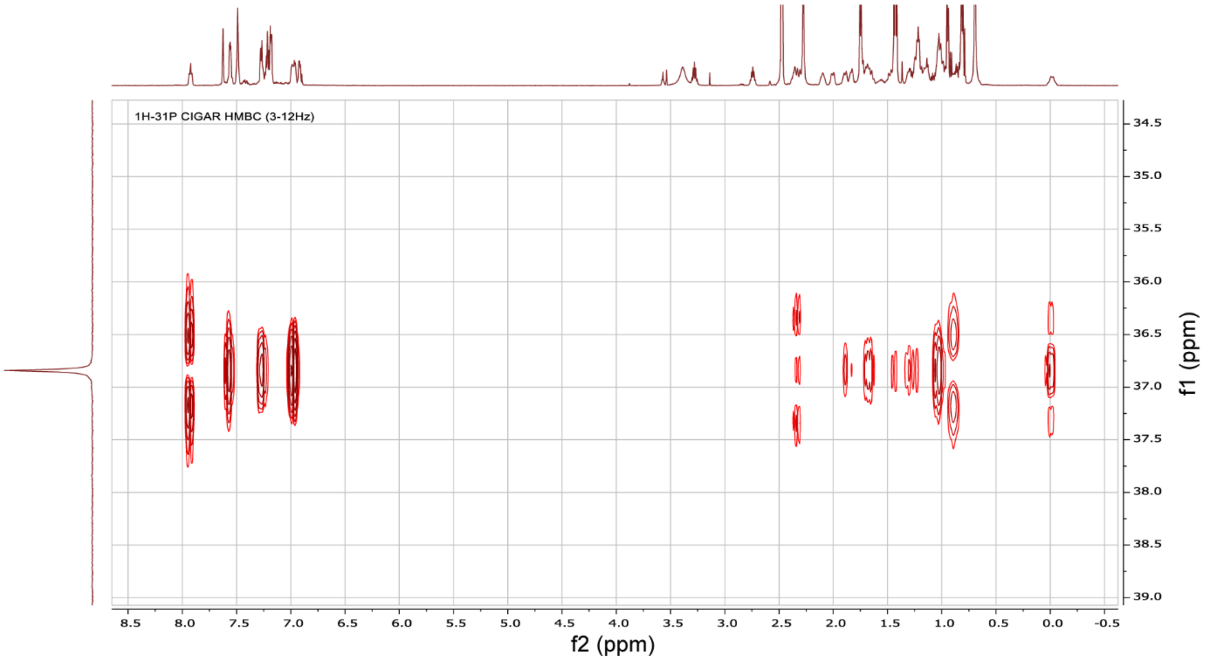

Figure S9. ^{1}H-^{31}P CIGAR of XPhos Pd G3 in DMSO-d_6 solution.

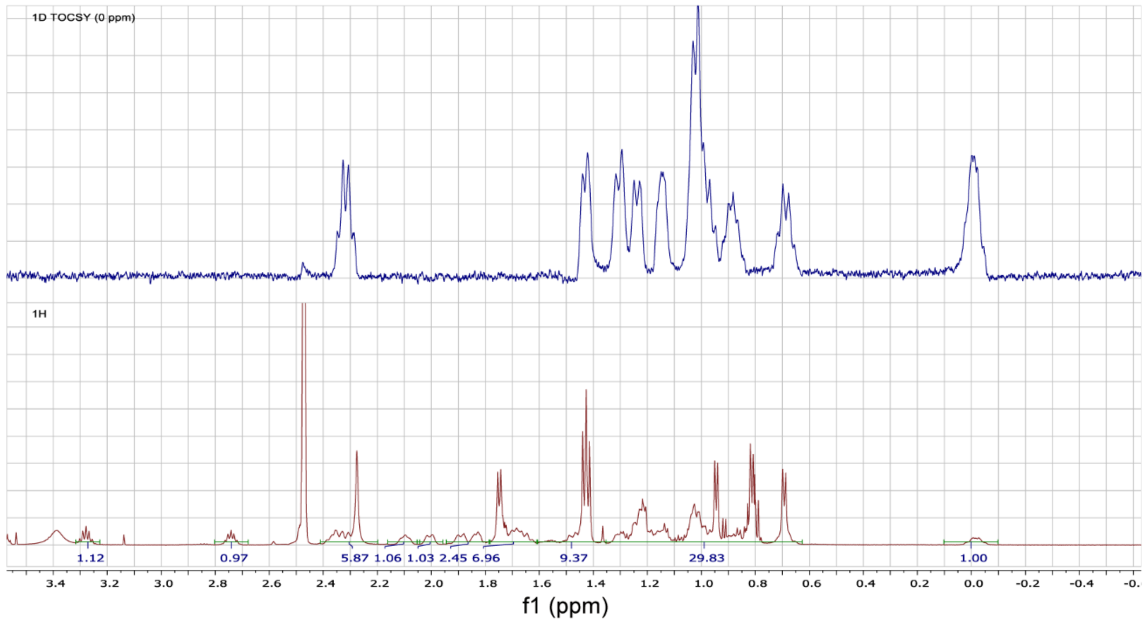

Figure S10. TOCSY of XPhos Pd G3 in DMSO-d_6 solution.

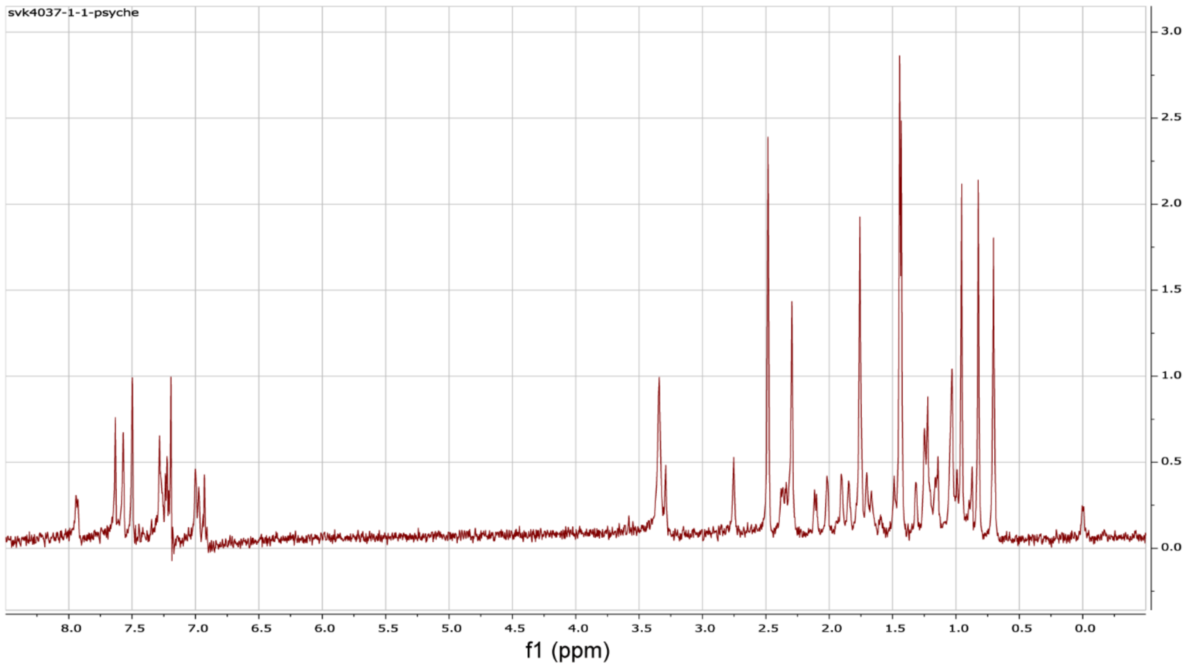

Figure S11. PSYCHE of XPhos Pd G3 in DMSO-d_6 solution.

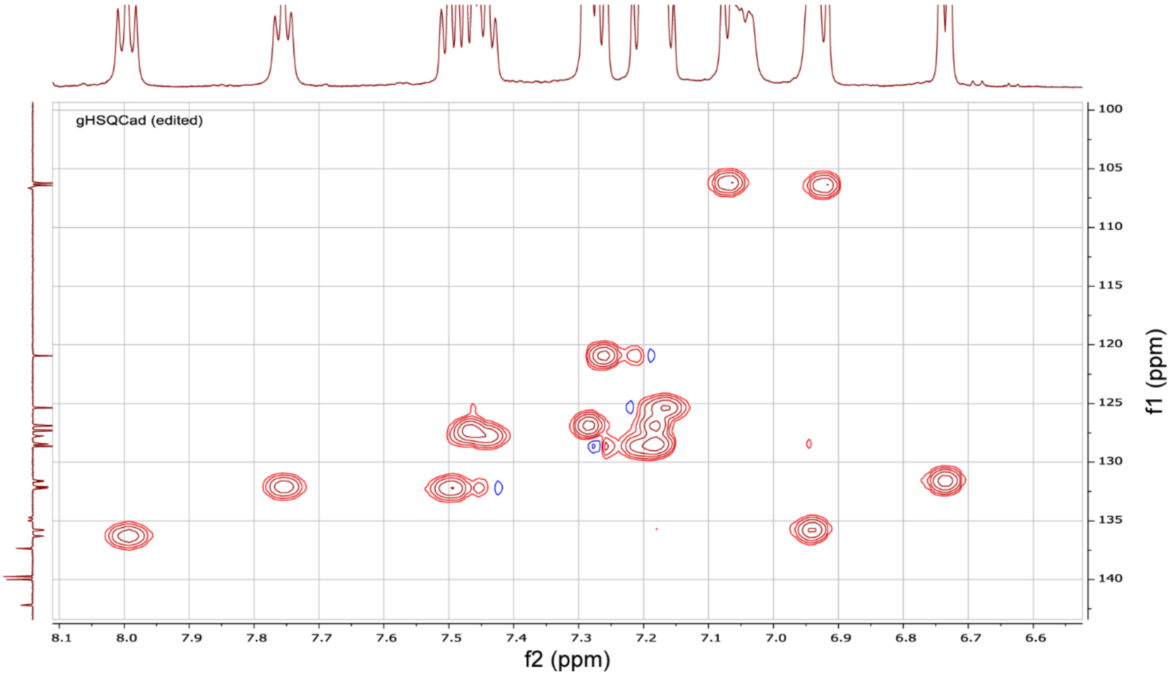

Figure S12. ^{1}H-^{13}C HMBC of RuPhos Pd G3 in DMSO-d_6 solution.

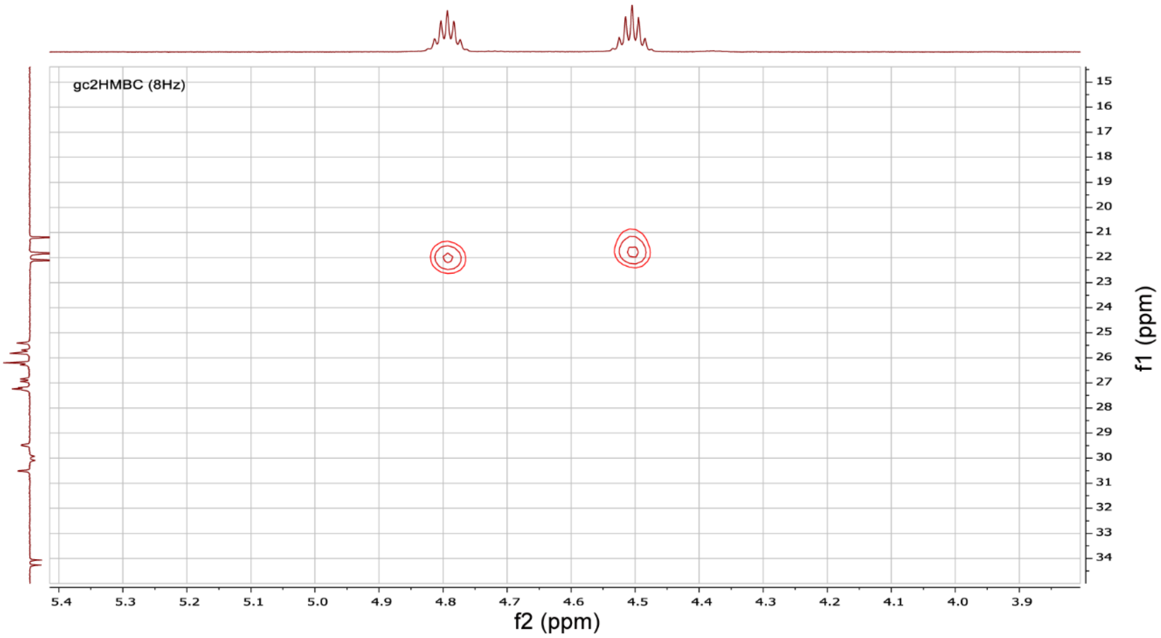

Figure S13. ^{1}H-^{13}C HMBC of RuPhos Pd G3 in DMSO-d_6 solution.

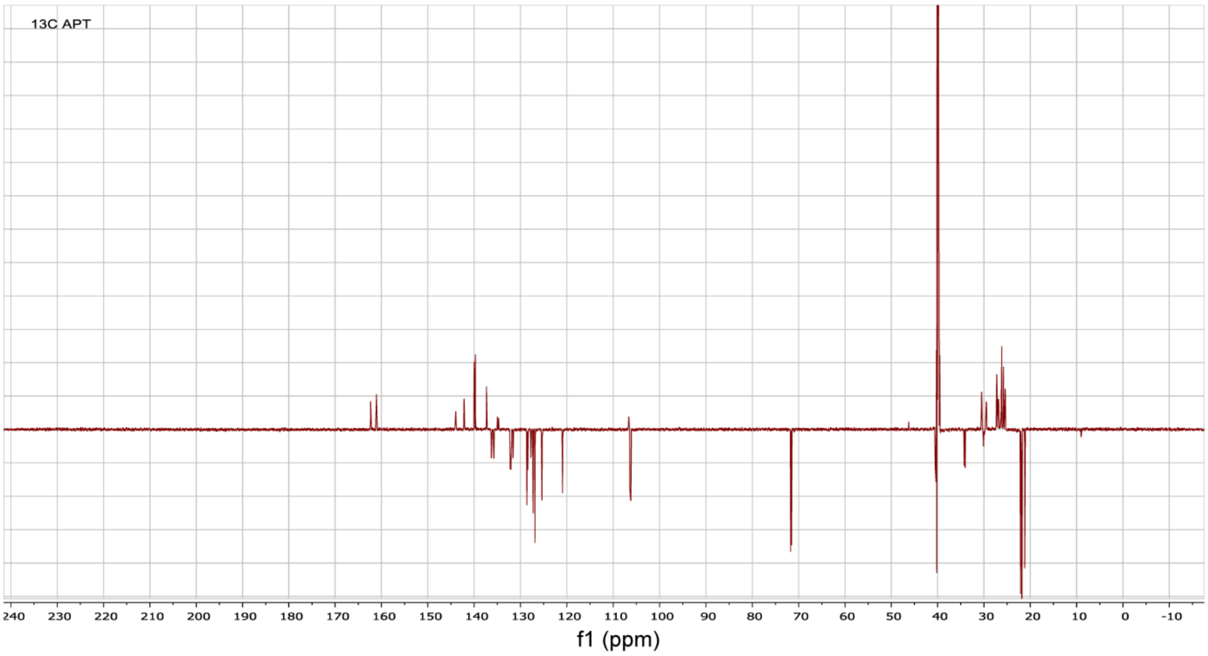

Figure S14. ^{13}C APT spectra of RuPhos Pd G3 in DMSO-d_6 solution.

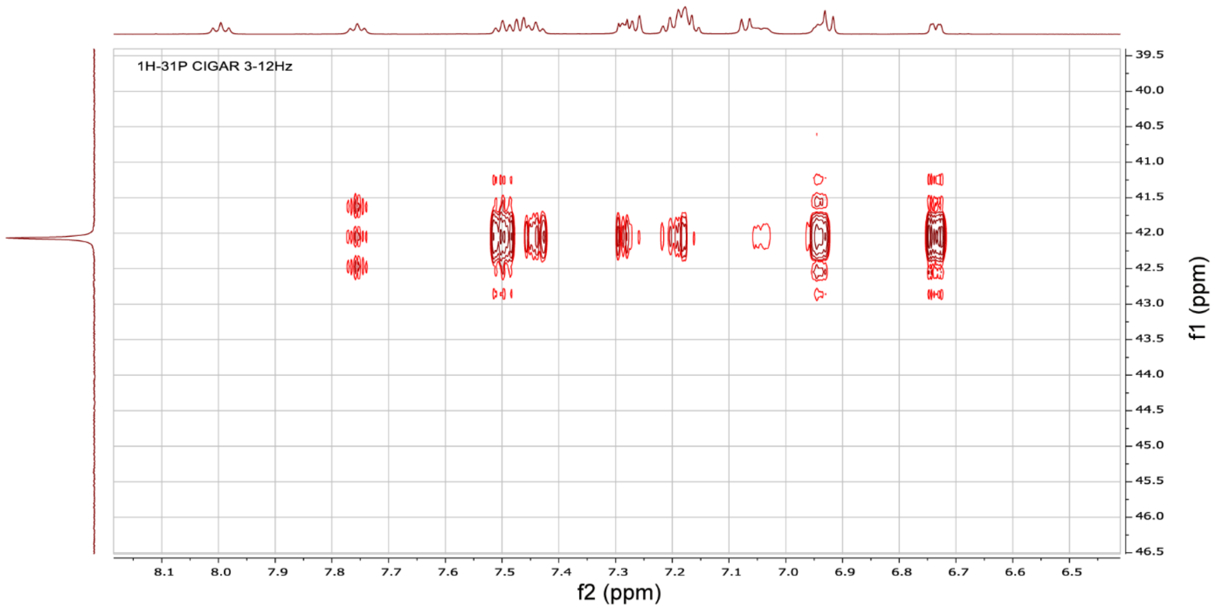

Figure S15. ^{1}H-^{31}P CIGAR of RuPhos Pd G3 in DMSO-d_6 solution.

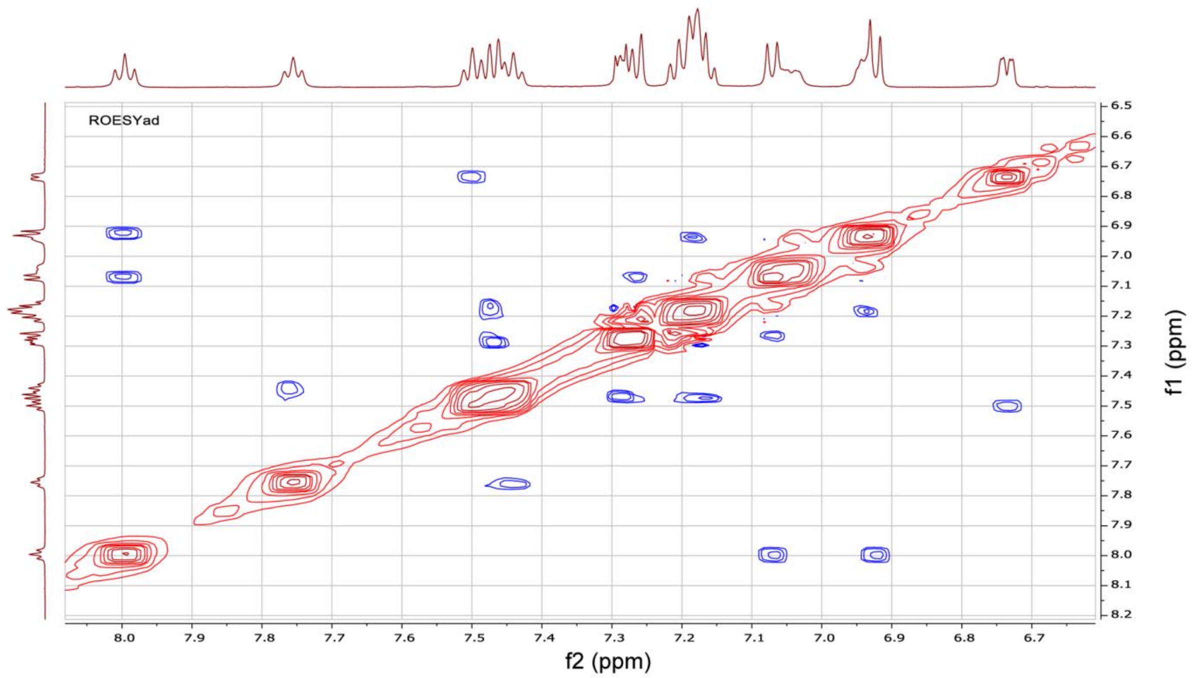

Figure S16. ROESY spectra of RuPhos Pd G3 in DMSO-d_6 solution.

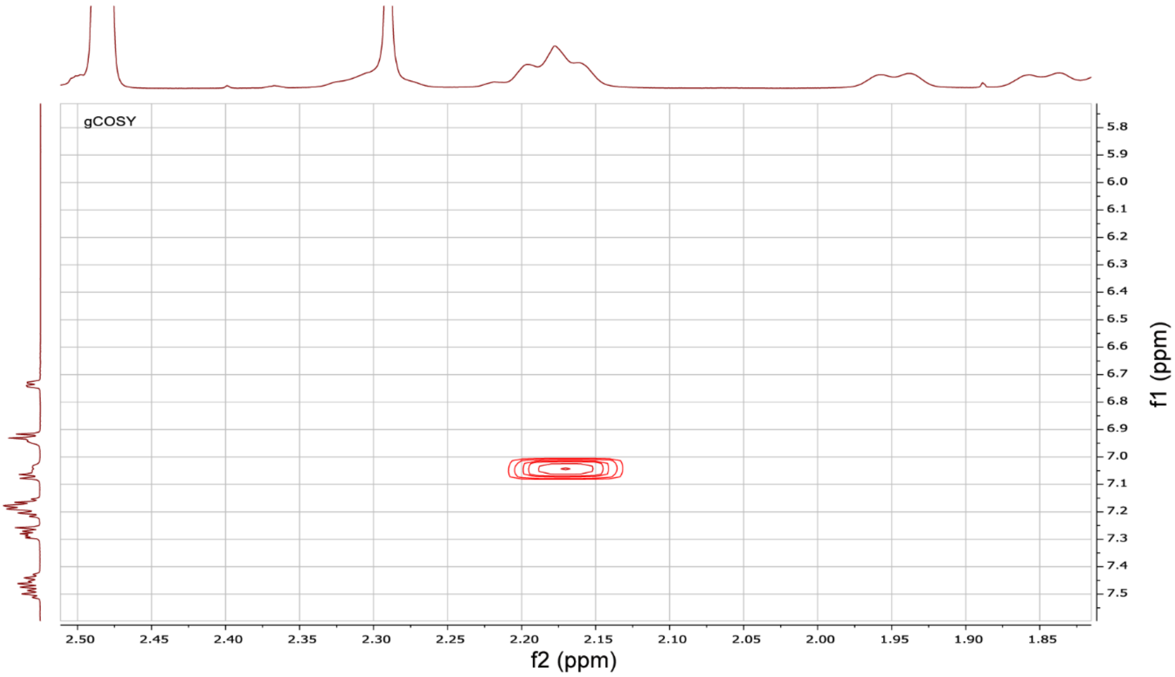

Figure S17. COSY of RuPhos Pd G3 in DMSO-d_6 solution.

Table S1. Formal analysis of ^{1}H NMR spectrum of XPhos Pd G3 used for assessment of impurity content

DMSO-d_6			CD_3OD	
Multiplet range, ppm	Notation	I_{theor}	Multiplet range, ppm	I_{theor}
8.01–7.89	A	1	7.99–7.88	1
7.69–7.46		5	7.63–7.45	5
7.36–7.15	B	5	7.33–7.15	4
7.06–6.90		3	7.13–7.05	1
Total number of aromatic protons		*14*	7.01–6.86	3
3.32–3.23		1	*Total number of aromatic protons*	*14*
2.83–2.70	H	1	3.39–3.31	1
2.44–2.23	D	4	2.96–2.83	1
2.17–1.97		2	2.27–2.61	3
1.97–1.82		2	2.58–2.47	1
1.82–1.61	E	6	2.38–2.20	2
1.57–1.38	I	8	2.13–2.03	1
1.37–1.12	F	6	2.03–1.72	8
1.12–0.78	G	13	1.64–1.46	8
0.78–0.64		4	1.46–0.95	15
0.08– -0.08		1	0.95–0.79	4
Total number of aliphatic protons		*48*	0.79–0.67	3
			0.23–0.06	1
			Total number of aliphatic protons	*48*

Table S2. Formal analysis of ^{1}H NMR spectrum of RuPhos Pd G3 used for assessment of impurity content

DMSO-d_6			CD_3OD	
Multiplet range, ppm	Notation	I_{theor}	Multiplet range, ppm	I_{theor}
8.04–7.96	A	1	8.05–7.98	1
7.81–7.73		1	7.82–7.76	1
7.55–7.42		3	7.58–7.44	3
7.33–7.14	B	5	7.36–7.16	6
7.11–7.03		2	7.12–7.03	2
6.98–6.91		2	6.95–6.88	1
6.78–6.73		1	6.81–6.74	1
Total number of aromatic protons		*15*	*Total number of aromatic protons*	*15*
4.85–4.76		1	4.84–4.77	1
4.56–4.47		1	4.61–4.53	1
2.33–2.26	D	3	2.77–2.65	3
2.24–2.14		3	2.53–2.43	1
2.00–1.92		1	2.41–2.32	1
1.90–1.78		2	2.23–2.12	1
1.78–1.67	E	1	2.08–1.90	3
1.54–1.36		6	1.90–1.78	1
1.34–0.90		14	1.76–1.66	1
0.82–0.66	F	5	1.64–1.46	5
0.66–0.58	G	3	1.46–0.96	14
-0.1– -0.21		1	0.95–0.77	5
Total number of aliphatic protons		*41*	0.76–0.69	3
			0.08– -0.04	1
			Total number of aliphatic protons	*41*
